# On-Site Genomic Epidemiological Analysis of Antimicrobial-Resistant Bacteria in Cambodia With Portable Laboratory Equipment

**DOI:** 10.3389/fmicb.2021.675463

**Published:** 2021-05-13

**Authors:** Aki Hirabayashi, Hideji Yanagisawa, Hiromizu Takahashi, Koji Yahara, Philipp Boeing, Bethan Wolfenden, Vandarith Nov, Vichet Lorn, Mom Veng, Vuth Ann, Chau Darapheak, Keigo Shibayama, Masato Suzuki

**Affiliations:** ^1^AMR Research Center, National Institute of Infectious Diseases, Tokyo, Japan; ^2^MicroSKY Lab., Inc., Tokyo, Japan; ^3^Department of General Medicine, Juntendo University School of Medicine, Tokyo, Japan; ^4^Bento Bioworks Ltd., London, United Kingdom; ^5^National Institute of Public Health, Phnom Penh, Cambodia; ^6^Department of Bacteriology II, National Institute of Infectious Diseases, Tokyo, Japan

**Keywords:** AMR, carbapenemase, CPE, Acinetobacter, Cambodia

## Abstract

The rapid emergence of carbapenemase-producing gram-negative bacteria (CPGNB) is a global threat due to the high mortality of infection and limited treatment options. Although there have been many reports of CPGNB isolated from Southeast Asian countries, to date there has been no genetic analysis of CPGNB isolated from Cambodia. Sequence-based molecular epidemiological analysis enables a better understanding of the genotypic characteristics and epidemiological significance of antimicrobial-resistant (AMR) bacteria in each country, and allows countries to enact measures related to AMR issues. In this study, we performed on-site genomic epidemiological analysis of CPGNB isolated in Cambodia using a portable laboratory equipment called Bento Lab, which combines a PCR thermal cycler, microcentrifuge, gel electrophoresis apparatus, and LED transilluminator, along with the MinION nanopore sequencer. PCR targeting of major carbapenemase genes using Bento Lab revealed that two *Escherichia coli* isolates and one *Acinetobacter baumannii* isolate harbored carbapenemase genes: *bla*_NDM_, *bla*_OXA–48_, and *bla*_OXA–23_, respectively. The results of phenotypic diagnostic tests for CPGNB, such as the carbapenem inactivation method and double-disk diffusion test using a specific inhibitor of metallo-β-lactamases, were consistent with their AMR genotypes. Whole-genome sequencing analysis using MinION revealed that *bla*_NDM–5_ gene was carried on a 93.9-kb plasmid with IncFIA/IncFIB/IncFII/IncQ1 replicons, and *bla*_OXA–181_ gene was carried on a 51.5-kb plasmid with the IncX3 replicon in *E. coli* isolates. *bla*_OXA–23_ was encoded in two locations on the chromosome of *A. baumannii*. Plasmids carrying *bla*_NDM–5_ or *bla*_OXA–181_ in *E. coli* were highly structurally identical to plasmids prevalent in Enterobacterales in China and other countries, suggesting that they disseminated from a common evolutionary origin. Our findings demonstrate the potential impact of portable laboratory equipment on AMR bacteria research in hospitals and research centers with limited research facilities, and provide the first glimpse into the genomic epidemiology of CPGNB in Cambodia.

## Introduction

Antimicrobial-resistant (AMR) bacteria have emerged and spread all over the world. Among antimicrobials, carbapenems are one of the most reliable last-resort antimicrobials for infections caused by AMR gram-negative bacteria. One of the mechanisms of AMR is drug inactivation mediated by acquired enzymes, such as β-lactamases (Santajit and Indrawattana, 2016). Among β-lactamases, extended-spectrum β-lactamases (ESBLs) and carbapenem-hydrolyzing β-lactamase (carbapenemases) are clinically important, as ESBLs hydrolyze a broad range of β-lactams, including cephalosporins, and carbapenemases hydrolyze most β-lactams, including carbapenems. β-lactamases are classified using the Ambler scheme as follows. Ambler class A includes ESBLs, such as CTX-M, as well as carbapenemases, such as KPC; class B includes metallo-β-lactamases (MBLs), such as NDM, IMP, and VIM; class C includes AmpC β-lactamases; and class D includes carbapenem-hydrolyzing oxacillinases, of which OXA-48 is prevalent in Enterobacterales, and OXA-23, OXA-24, and OXA-58 are prevalent in *Acinetobacter* spp. ESBL and carbapenemase genes are predominantly encoded on conjugative plasmids and have been transferred among Enterobacterales and other gram-negative bacteria (Tzouvelekis et al., 2012). Moreover, carbapenemase-producing gram-negative bacteria (CPGNB) harboring carbapenemase genes often co-harbor clinically relevant antimicrobial resistance genes, such as aminoglycoside and fluoroquinolone resistance genes (Wu et al., 2007; Chmelnitsky et al., 2008; Poirel et al., 2011a). There is great concern about the global spread of plasmids that carry multiple AMR genes that can be transferred between homogeneous and heterogeneous species.

Although CPGNB has been detected in large numbers in Southeast Asia, the publicly available information on CPGNB is limited to a small number of countries (Malchione et al., 2019). In 2015, the World Health Organization (WHO) adopted a global action plan on AMR and launched the Global Antimicrobial Resistance Surveillance System (GLASS), the first global collaborative report to standardize AMR surveillance (WHO, 2015; Tornimbene et al., 2018). Cambodia’s Laboratory Information System (CamLIS) was developed by the Ministry of Health in Cambodia with the support of WHO starting in 2011 (WHO, 2019). As of 2018, 35 national, provincial, and referral laboratories contribute to CamLIS. Cambodia has started to prepare the GLASS report for 2020, and the actual status of AMR bacteria in the country will be revealed in the near future. To date, however, only a few studies have examined AMR bacteria clinically isolated in Cambodia, although other Southeast Asian countries are increasingly reporting cases of AMR bacteria (Suwantarat and Carroll, 2016; Gandra et al., 2020). To date, there has been no report on the genomic epidemiology of CPGNB in Cambodia.

In this study, we introduced portable laboratory equipment, Bento Lab and MinION, for on-site genomic epidemiological analysis of CPGNB in Cambodia. Bento Lab (Bento Bioworks Ltd., United Kingdom) is a DNA analysis device small enough to fit in a laptop-sized bag. It contains a PCR thermal cycler, microcentrifuge, and gel electrophoresis apparatus with LED transilluminator, and has sufficient functionality for laboratory work (Bento Lab, 2016). The MinION nanopore sequencer (Oxford Nanopore Technologies, United Kingdom) is a portable long-read sequencer with the size of a large USB memory stick. MinION was utilized for on-site genomic epidemiological analysis of the Ebola virus outbreak in West Africa in 2016 (Quick et al., 2016) and the Zika virus outbreak in the Americas in 2017 (Faria et al., 2017). Because carbapenemase genes are mostly carried on plasmids, long-read sequencing is useful for assembling whole plasmid sequences and tracking horizontal transfer of AMR plasmids in hospitals, as well as local and global communities (Conlan et al., 2014).

We organized an international collaborative research group with researchers from Japan, United Kingdom, and Cambodia, and successfully performed on-site genomic epidemiological analysis of CPGNB clinical isolates in Cambodia, where access to laboratory equipment is limited. Our findings demonstrate the potential impact of portable laboratory equipment on AMR bacteria research and provide the first glimpse into the genomic epidemiology of CPGNB in Cambodia.

## Materials and Methods

### Ethics

Written informed consent was obtained from the individuals for the publication of any potentially identifiable images included in this article.

### Subjects and Specimen Collection

The outpatient clinic of National Institute of Public Health (NIPH) in Phnom Penh, Cambodia has around 10 patients in a day and 456 bacterial strains were isolated from patient specimens, such as sputum, stool, urine, pus, body fluid, and cerebral spinal fluid, in 2017. Ethical approval of this study “Genomic epidemiological analysis of AMR bacterial isolates in Cambodia” was obtained from National Ethics Committee for Health Research (NECHR), Cambodia (approval no.: 178NECHR). Two carbapenemase-producing isolates NIPH17_0020 and NIPH17_0036 of *Escherichia coli* and one carbapenemase-producing isolate NIPH17_0019 of *Acinetobacter baumannii* analyzed in this study were obtained from abdominal pus, urine, and blood of patients, respectively, at NIPH, Cambodia in 2017.

### Bacterial Isolates

Bacterial species identification was performed using conventional biochemical tests (e.g., citrate test, urease test, hydrogen sulfide test, oxidase test, indole test, lysine decarboxylase test, and carbohydrate fermentation test) and the API 20E/20NE systems (bioMérieux), and antimicrobial susceptibility testing with *E. coli* ATCC 25922 as quality control was performed using BBL Sensi-Disc Susceptibility Test Disks (BD) as part of routine diagnosis at NIPH, Cambodia. Minimum inhibitory concentrations (MICs) of selected antimicrobials, including imipenem (IPM), meropenem (MEM), ceftazidime (CAZ), cefotaxime (CTX), aztreonam (AZT), amikacin (AMK), and ciprofloxacin (CPFX), against carbapenemase-producing isolates of *E. coli* (NIPH17_0020 and NIPH17_0036) and *A. baumannii* (NIPH17_0019) were further examined using the E-test strips (bioMérieux) in this study. The breakpoints for susceptible (S), intermediate (I), and resistance (R) to antimicrobials were adopted from the Clinical and Laboratory Standards Institute (CLSI) 2020 guidelines. Carbapenemase production was examined using the carbapenem inactivation method (CIM) according to the CLSI guidelines also as routine diagnosis at NIPH, Cambodia. The double-disk diffusion tests (DDDTs) with clavulanate (CVA) and sodium mercaptoacetic acid (SMA) as specific inhibitors for extended spectrum β-lactamases (ESBLs) and metallo-β-lactamases (MBLs), respectively, were performed as previously described (Arakawa et al., 2000; Hattori et al., 2013; CLSI, 2015). Briefly, the production of ESBLs was tested with the combination of CAZ, CTX, and clavulanate/amoxicillin (CVA/AMPC) disks (Eiken Chemical Co.), and production of MBLs was tested with the combination of IPM and SMA disks (Eiken Chemical Co.).

### PCR, Whole-Genome Sequencing, and Bioinformatics Analysis

Draft genome analysis of carbapenemase-producing isolates of *E. coli* (NIPH17_0020 and NIPH17_0036) and *A. baumannii* (NIPH17_0019) using Bento Lab and MinION was performed in NIPH, Cambodia in July, 2017. Bacterial genomic DNAs (gDNAs) were extracted using the MagAttract HMW DNA Kit (Qiagen) and quantified using a Qubit 2.0 fluorometer (Thermo Fisher Scientific). Library preparation for MinION sequencing using Rapid Sequencing Kits (SQK-RAD002 and SQK-RAD003) (Oxford Nanopore Technologies) were performed using the prototype model of Bento Lab (Bento Bioworks Ltd.) consisting of a thermal cycler, microcentrifuge, and gel electrophoresis apparatus with LED transilluminator (Bento Lab, 2016). The prototype is configured slightly differently from the current commercial versions, but there is no significant difference in performance (Supplementary Figure 1).

PCR for selected carbapenemase genes, *bla*_NDM_ (621-bp), *bla*_KPC_ (798-bp), *bla*_IMP_ (232-bp), *bla*_VIM_ (390-bp), *bla*_OXA–48_ (438-bp), *bla*_OXA–23_ (501-bp), *bla*_OXA–24_ (246-bp), *bla*_OXA–51_ (353-bp), and *bla*_OXA–58_ (599-bp)] was performed using primers as previously described (Woodford et al., 2006; Poirel et al., 2011b). PCR amplification products were subjected to agarose gel electrophoresis using electrophoresis apparatus supplied with Bento Lab, stained with SYBR Gold Nucleic Acid Gel Stain (Thermo Fisher Scientific), detected using LED transilluminator built in Bento Lab, and photographed with iPhone 7 Plus (Apple).

Whole-genome sequencing was performed on the MinION nanopore sequencer (Oxford Nanopore Technologies) for 24 h with the offline-capable version of MinKNOW v1.7.3 and R9.4 flow cells according to the manufacturer’s instructions. Nanopore reads were base called using Albacore v2.1.0 (Oxford Nanopore Technologies), corrected using Genome Finishing Module v1.7 plugged-in CLC Genomics Workbench v10.1.1 (Qiagen) with default parameters of Correct PacBio Reads (beta), and assembled *de novo* using Miniasm v0.2 (Li, 2016) with default parameters.

The extracted bacterial gDNAs were subsequently re-sequenced on a Illumina system in National Institute of Infectious Diseases, Japan for further error correction. Library for Illumina sequencing (insert size of 500–900 bp) was prepared using Nextera XT DNA Library Prep Kit (Illumina) and paired-end sequencing (2 bp× 150 bp) was performed using MiniSeq (Illumina). Illumina paired-end reads were mapped onto the on-site assembly sequences, and sequencing errors were corrected by extracting the consensus of the mapped reads five times using CLC Genomics Workbench v12.0 (Qiagen) with default parameters.

Genome sequences were annotated using the DFAST server^1^. Sequence type (ST), plasmid replicon type, AMR genes, and virulence genes were detected using MLST v2.0, PlasmidFinder v2.1, ResFinder v4.1, and VirulenceFinder v2.0, respectively, using the CGE server^2^ with default parameters. Type IV secretion system (T4SS)-associated genes involved in conjugation were detected using TXSScan^3^ with default parameters. Mobile gene elements (MGEs) were identified manually from CDS annotations and basically analyzed by comparing the sequences analyzed in previous studies. Linear comparisons of sequences carrying carbapenemase genes were performed using BLAST with default settings (the nucleotide collection database and the megablast program) and visualized using Easyfig v2.2.2^4^. The annotated bacterial circular chromosomes were visualized using the CGView Server^5^.

Genome and plasmid sequences of carbapenemase-producing *E. coli* (NIPH17_0020 and NIPH17_0036) and *A. baumannii* (NIPH17_0019) isolated in Cambodia have been deposited at GenBank/EMBL/DDBJ under accession numbers AP024560 (NIPH17_0020), LC483178 (pNIPH17_0020_1), AP024561 (NIPH17_0036), LC483179 (pNIPH17_0036_1), LC603215 (pNIPH17_0036_2), and AP024415 (NIPH17_0019).

## Results

### On-Site Genomic Epidemiological Analysis of Carbapenemase-Producing Gram-Negative Bacteria Isolated in Cambodia

**FIGURE 1:**
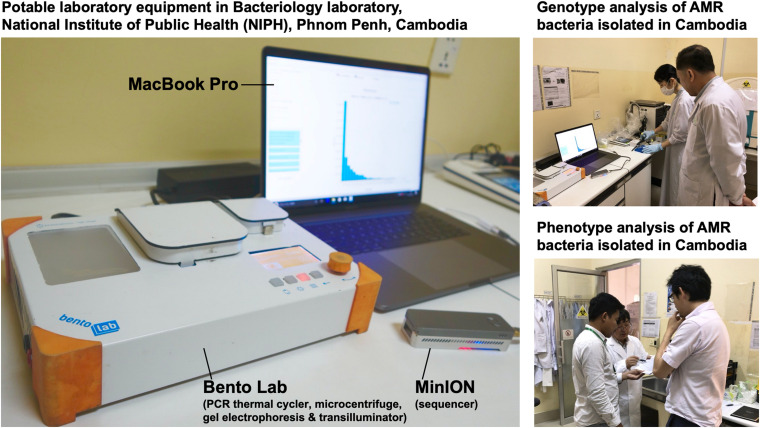
On-site genomic epidemiological analysis of AMR bacteria in Cambodia. Bento Lab and MinION were used for genotype analysis and the carbapenem inactivation method (CIM) and double-disk diffusion tests (DDDTs) were used for phenotype analysis of AMR bacteria.

**FIGURE 2:**
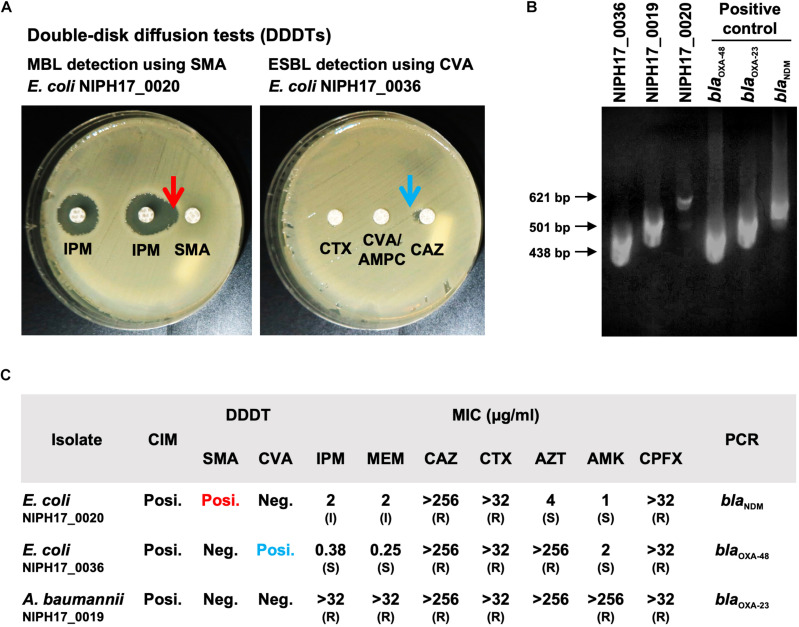
Genotype and phenotype analysis of AMR bacteria isolated in Cambodia. **(A)** The double-disk diffusion test (DDDT) with imipenem (IPM) and sodium mercaptoacetic acid (SMA) disks against *E. coli* NIPH17_0020 for MBL detection and DDDT with ceftazidime (CAZ), cefotaxime (CTX), and clavulanate/amoxicillin (CVA/AMPC) disks against *E. coli* NIPH17_0036 for ESBL detection. Arrows indicate β-lactamase inhibition. **(B)** PCR amplifications for *bla*_OXA–48_ for *E. coli* NIPH17_0036 (438 bp), *bla*_OXA–23_ for *A. baumannii* NIPH17_0019 (501 bp), *bla*_NDM_ for *E. coli* NIPH17_0020 (621 bp) and their positive controls. Some of the images are unclear because they were acquired in a location with limited equipment. **(C)** Summary of genotype and phenotype analysis, including the carbapenem inactivation method (CIM), DDDTs with SMA or CVA, minimum inhibitory concentrations (MIC) measurement, and PCR targeting selected major carbapenemase genes. The breakpoints for susceptible (S), intermediate (I), and resistance (R) to antimicrobials were adopted from the CLSI 2020 guidelines.

**FIGURE 3:**
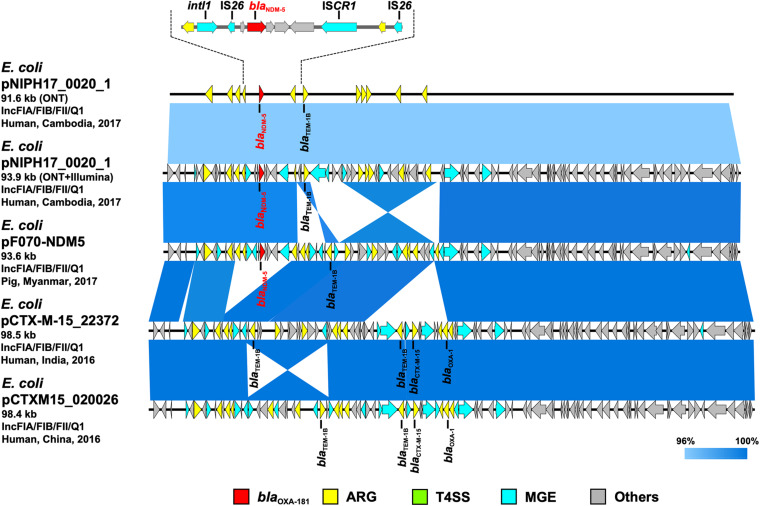
Linear comparison of related plasmid sequences from Cambodia and other countries. *E. coli* pNIPH17_0020_1 with *bla*_NDM–5_ (this study, accession no. LC483178), *E. coli* pF070-NDM5 with *bla*_NDM–5_ (accession no. AP023238), *E. coli* pCTX-M-15_22372 with no *bla*_NDM_ (accession no. CP040398), and *E. coli* pCTXM15_020026 with no *bla*_NDM_ (accession no. CP034956) are shown. Regarding *E. coli* pNIPH17_0020_1, both the uncorrected sequence (from on-site ONT analysis) and corrected sequence (from subsequent ONT + Illumina analysis), and the detailed genetic structures around *bla*_NDM–5_ are shown (upper). Red, yellow, green, blue, and gray arrows indicate carbapenemase gene (*bla*_NDM–5_), other AMR genes (ARG), type IV secretion system-associated genes involved in conjugation (T4SS), mobile gene elements (MGE), and other genes (Others), respectively. The colors in comparison of plasmids show percent identity.

**FIGURE 4:**
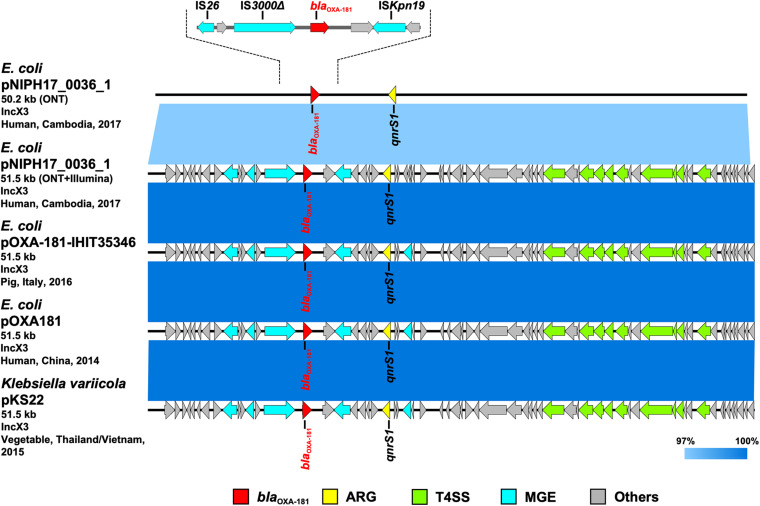
Linear comparison of related plasmid sequences from Cambodia and other countries. *E. coli* pNIPH17_0036_1 with *bla*_OXA–181_ (this study, accession no. LC483179), *E. coli* pOXA-181-IHIT35346 with *bla*_OXA–181_ (accession no. KX894452), *E. coli* pOXA-181 with *bla*_OXA–181_ (accession no. KP400525), and *Klebsiella variicola* pKS22 with *bla*_OXA–181_ (accession no. KT005457) are shown. Regarding *E. coli* pNIPH17_0036_1, both the uncorrected sequence (from on-site ONT analysis) and corrected sequence (from subsequent ONT + Illumina analysis), and the detailed genetic structures around *bla*_OXA–181_ are shown (upper). Red, yellow, green, blue, and gray arrows indicate carbapenemase gene (*bla*_OXA–181_), other AMR genes (ARG), type IV secretion system-associated genes involved in conjugation (T4SS), mobile gene elements (MGE), and other genes (Others), respectively. The colors in comparison of plasmids show percent identity.

**FIGURE 5:**
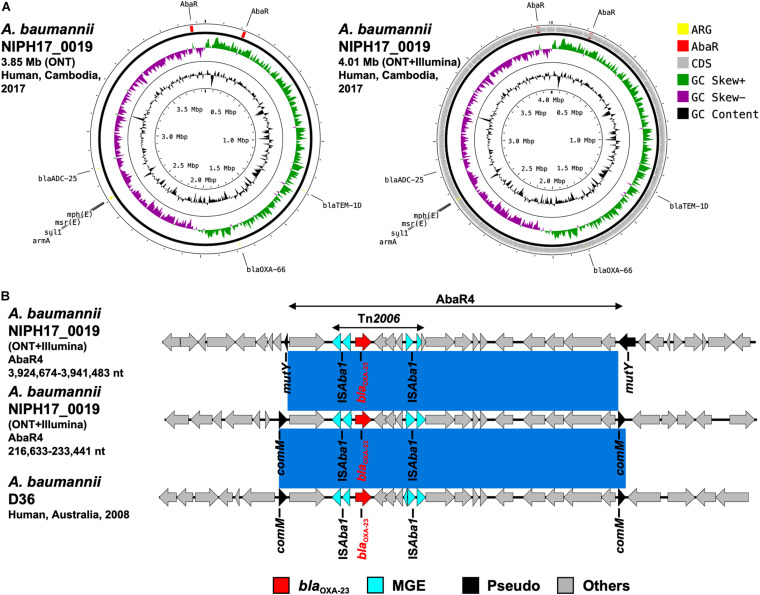
**(A)** Circular chromosome representation of *A. baumannii* NIPH17_0019 harboring two copies of *bla*_OXA–23_. Both the uncorrected sequence (from on-site ONT analysis) and corrected sequence (from subsequent ONT + Illumina analysis, accession no. AP024415) are shown. Yellow, red, gray, green, purple, and black indicate AMR genes (ARG), AbaR4 with *bla*_OXA–23_ (AbaR), other coding sequences (CDS), GC skew+, GC skew-, and GC content, respectively. **(B)** Linear comparison of AbaR4-containing genomic regions in *A. baumannii* from Cambodia and other countries. Two sets of AbaR4 with *bla*_OXA–23_ in *A. baumannii* NIPH17_0019 (3,924,674–3,941,483 nt region inserted within *mutY* and 216,633–233,441 nt region inserted within *comM* in accession no. AP024415) and AbaR4 with *bla*_OXA–23_ in *A. baumannii* D36 (41,312–58,123 nt region inserted within *comM* in accession no. JN107991) are shown. Red, blue, black, and gray arrows indicate carbapenemase gene (*bla*_OXA–23_), mobile gene elements (MGE), pseudogenes disrupted by AbaR4 insertions (Pseudo), and other genes (Others), respectively. The blue color in comparison of sequences indicates nearly 100% identity.

In July 2017, we stayed for 5 days at the National Institute of Public Health (NIPH) in Phnom Penh, Cambodia. There, we set up potable laboratory equipment, including Bento Lab and MinION, in the Bacteriology laboratory, which has limited research facilities and no PCR machine (Figure 1). On the first and second days, we performed the carbapenem inactivation method (CIM) test, double-disk diffusion tests (DDDTs), minimum inhibitory concentrations (MICs) measurement, PCR, and MinION sequencing on two carbapenemase-producing isolates of *E. coli* (NIPH17_0020 and NIPH17_0036) and one carbapenemase-producing isolate of *A. baumannii* (NIPH17_0019) (Figure 1) stored in the laboratory prior to this study. On the third and fourth days, we examined diagnostic testing data (Figure 2) and analyzed sequencing data (Figures 3–5). On the final day, we discussed the results of genotype and phenotype analysis with researchers and technicians belonging to the Bacteriology laboratory at NIPH.

The CIM test is routinely performed at NIPH, and we confirmed that all three bacterial isolates were positive for carbapenemase production. DDDTs with sodium mercaptoacetic acid (SMA) and clavulanic acid (CVA) disks revealed that *E. coli* NIPH17_0020 and *E. coli* NIPH17_0036 were positive for MBL and ESBL production, respectively (Figure 2A). PCR targeting major carbapenemase genes revealed that *E. coli* NIPH17_0020, *E. coli* NIPH17_0036, and *A. baumannii* NIPH17_0019 were positive for *bla*_NDM_, *bla*_OXA–48_, and *bla*_OXA–23_, respectively (Figure 2B). The MICs of imipenem (IPM) and meropenem (MEM) against *E. coli* NIPH17_0020, *E. coli* NIPH17_0036, and *A. baumannii* NIPH17_0019 were 2 (I) and 2 (I), 0.38 (S) and 0.25 (S), and > 32 (R) and > 32 μg/mL (R), respectively. Furthermore, the MICs of ceftazidime (CAZ), cefotaxime (CTX), aztreonam (AZT), amikacin (AMK), and ciprofloxacin (CPFX) against *E. coli* NIPH17_0020 were >256 (R), >32 (R), 4 (S), 1 (S), and >32 μg/mL (R), respectively; those against *E. coli* NIPH17_0036 were >256 (R), >32 (R), >256 (R), 2 (S), and >32 μg/mL (R), respectively; and those against *A. baumannii* NIPH17_0019 were >256 (R), >32 (R), >256, >256 (R), and >32 μg/mL (R), respectively (Figure 2C).

Based on the on-site *de novo* assembly sequences obtained from nanopore sequencing analysis, we determined the complete structures of chromosomes and plasmids of each bacterial isolate and detected AMR genes. As shown in Supplementary Table 1, *E. coli* NIPH17_0020 had two contigs (the 4.78-Mb chromosome and the 91.6-kb plasmid pNIPH17_0020_1); *E. coli* NIPH17_0036 had three contigs (the 4.68-Mb chromosome, the 50.2-kb plasmid pNIPH17_0036_1, and the 92.6-kb plasmid pNIPH17_0036_2); and *A. baumannii* NIPH17_0019 had only one contig (the 3.85-Mb chromosome). *E. coli* pNIPH17_0020_1 carried the *bla*_NDM–5_-like gene (96.3% identity and 3.0% gap relative to *bla*_NDM–5_: accession no. JN104597) (Supplementary Figure 2A); *E. coli* pNIPH17_0036_1 carried the *bla*_OXA–48_ family carbapenemase *bla*_OXA–181_-like gene (97.2% identity and 2.5% gap relative to *bla*_OXA–181_: accession no. CM004561) (Supplementary Figure 2B); and *A. baumannii* NIPH17_0019 harbored *bla*_OXA–23_-like genes in two separate locations of the chromosome (97.7% identity and 2.1% gap or 93.9 and 5.6% gap relative to *bla*_OXA–23_: accession no. AY795964) (Supplementary Figure 2C).

To summarize the results, the detected carbapenemase genes had a few-percent mismatch that caused frameshifts of genes relative to their putative reference sequences (Supplementary Figure 2); hence, we avoided performing CDS annotation for the on-site sequences. We detected AMR genes and plasmid replicons in the sequences analyzed on-site by sequence-based detection (Figures 3–5 and Supplementary Table 1). AMR genes, such as *bla*_TEM–1B_, *aadA2*, *aph(3″)-lb*, *aac(3)-lld*, and *aph(6)-ld*, and plasmid replicons, including IncFIA, IncFIB, IncFII, and IncQ1, were detected in provisional *bla*_NDM–5_-carrying pNIPH17_0020_1 in *E. coli* NIPH17_0020 (Figure 3), and *qnrS1* genes and the IncX3 replicon were detected in provisional *bla*_OXA–181_-carrying pNIPH17_0036_1 in *E. coli* NIPH17_0036 (Figure 4). BLAST searches of *E. coli* pNIPH17_0020_1 and *E. coli* pNIPH17_0036_1 revealed that several plasmids from Asian and Western countries were highly identical to those plasmids (Figures 3, 4).

### Comparison of Plasmids and Genomic Regions in Carbapenemase-Producing Gram-Negative Bacteria Isolated in Cambodia With Those in Other Countries

After the on-site analysis in Cambodia, we further performed Illumina sequencing of carbapenemase-producing isolates of *E. coli* (NIPH17_0020 and NIPH17_0036) and *A. baumannii* (NIPH17_0019), corrected the on-site *de novo* assembly sequences using Illumina reads, and compared the on-site and error-corrected sequences (Figures 3, 4, 5A, and Supplementary Figure 3). The on-site sequences of *E. coli* pNIPH17_0020_1 (provisional 91.6-kb *bla*_NDM–5_-carrying plasmid with IncFIA/FIB/FII/Q1 replicons) and *E. coli* pNIPH17_0036_1 (provisional 50.2-kb *bla*_OXA–181_-carrying plasmid with IncX3 replicons) were highly identical to their error-corrected sequences (96.48% identity over 100% of the error-corrected 93.9-kb plasmid pNIPH17_0020_1: accession no. LC483178 and 96.70% identity over 100% of the error-corrected plasmid 51.5-kb pNIPH17_0036_1: accession no. LC483179, respectively) (Figures 3, 4). Moreover, the on-site sequences of *bla*_OXA–23_-containing chromosomal regions of *A. baumannii* NIPH17_0019 were also highly identical to their error-corrected sequences (96.95% identity over 100% of 216,633–233,441 nt and 97.36% identity over 100% of 3,924,674–3,941,483 nt in their error-corrected chromosome of NIPH17_0019: accession no. AP024415) (Supplementary Figures 3A,B). Although there were differences of a few percent between the on-site and error-corrected sequences, the best match types of AMR genes and plasmid replicons detected from the reference libraries were consistent (Figures 3, 4, 5A).

*Escherichia coli* NIPH17_0020 belonged to sequence type 410 (ST410) according to multilocus sequence typing (MLST) analysis and harbored putative virulence genes, including *fyuA*, *gad*, *irp2*, *lpfA*, and *terC* on its chromosome (Supplementary Table 1). NIPH17_0020 had one 93.9-kb plasmid, pNIPH17_0020_1, with a backbone consisting of IncFIA/FIB/FII/Q1 with multiple AMR genes, such as β-lactamase (*bla*_NDM–5_ and *bla*_TEM–1B_) and aminoglycoside resistance genes [*aac(3)-lld*-like, *aadA2, aph(3”)-lb, aph(6)-ld*] (Supplementary Table 1). *E. coli* pNIPH17_0020_1 was structurally highly identical to plasmid pPF070-NDM5 (accession no. AP023238) in *E. coli* isolated from a pig in Myanmar in 2017, plasmid pCTX-M-15_22372 (accession no. CP040398) in *E. coli* isolated from a human in India in 2016, and plasmid pCTXM15_020026 (accession no. CP034956) in *E. coli* isolated from a human in China in 2016 (99.8% identity over 92–94% of pNIPH17_0020_1) (Figure 3). pNIPH17_0020_1 contained several mobile gene elements (MGEs), including IS*26* and IS*CR1*, surrounding *bla*_NDM–5_ (Figure 3 upper). pF070-NDM5 carried *bla*_NDM–5_ with the same MGEs, whereas pCTX-M-15_22372 and pCTXM15_020026 did not carry *bla*_NDM–5_ (Figure 3).

*Escherichia coli* NIPH17_0036 also belonged to ST410 according to MLST analysis and harbored putative virulence genes, including *gad*, *hra*, *lpfA*, and *terC* on its chromosome (Supplementary Table 1). NIPH17_0036 had two plasmids; one of them, 51.5-kb IncX3 plasmid pNIPH17_0036_1 carrying AMR genes, including *bla*_OXA–181_ and *qnrS1* (Supplementary Table 1), was structurally nearly identical to plasmid pOXA-181-IHIT35346 (accession no. KX894452) in *E. coli* isolated from a pig in Italy in 2016 and plasmid pOXA181 (accession no. KP400525) in *E. coli* isolated from a human in China in 2014 (Liu et al., 2015), as well as plasmid pKS22 (accession no. KT005457) in *Klebsiella variicola* isolated from a fresh vegetable imported from Thailand or Vietnam (99.9% identity over 100% of pNIPH17_0036_1) (Figure 4). *bla*_OXA–181_ in pNIPH17_0036_1 was surrounded by several MGEs, including IS*Kpn19*, IS*3000*, and IS*26* (Figure 4 upper). *E. coli* NIPH17_0036 had another plasmid, pNIPH17_0036_2 (94.8-kb IncFIA/FIB/FII/Q1 plasmid, accession no. LC603215), carrying *bla*_CTX–M–15_, *bla*_OXA–1_, *bla*_TEM–1B_, other β-lactamase genes, and multiple aminoglycoside resistance genes (Supplementary Table 1).

*Acinetobacter baumannii* NIPH17_0019 belonged to ST471 according to MLST analysis and harbored the *bla*_OXA–23_ genes in two separate regions on the chromosome (accession no. AP024415) (Figure 5A). Both copies of *bla*_OXA–23_ were located in Tn*2006* in AbaR4. AbaR is the resistance island containing AMR genes in *A. baumannii* (Bi et al., 2019). Comparison of the genetic environment around AbaR4 was performed between *A. baumannii* NIPH17_0019 and AbaR4-harboring *A. baumannii* D36 (accession no. JN107991), which was isolated from a human in 2008 in Australia (Figure 5B). The results revealed that two AbaR4 islands in *A. baumannii* NIPH17_0019 were highly identical to that of *A. baumannii* D36 (99.84–99.88% identity over 100% of the sequence) (Figure 5B). Interestingly, AbaR4 was integrated into the *comM* gene in *A. baumannii* D36, whereas AbaR4 was integrated into the *comM* gene (AbaR4 of the 216,633–233,441 nt region) and *mutY* genes (for AbaR4 of the 3,924,674–3,941,483 nt region) in *A. baumannii* NIPH17_0019.

## Discussion

In this study, we successfully conducted genomic analysis of carbapenemase-producing gram-negative bacteria (CPGNB) in Cambodia and provided the first glimpse into the genomic epidemiology of CPGNB in that country. The main analysis was performed on-site using portable laboratory equipment, namely, Bento Lab and MinION. The nearly complete genomes of carbapenemase-producing *E. coli* and *A. baumannii* isolates, including their plasmids, were determined using the MinION nanopore sequencing data; detection of AMR genes and plasmid replicons, as well as sequence similarity searches in public databases, were performed at a Cambodian laboratory with limited research facilities. Because the nanopore sequencing technology is still evolving, and the accuracy of sequencing is imperfect (the rates of indels and substitutions are a few percent each) (Jain et al., 2016), it was necessary to combine other sequencing technologies, such as Illumina sequencing by synthesis, for further molecular typing analysis of bacteria that require more accurate sequences at the single-nucleotide level.

The cost of commercial versions of Bento Lab is starting from $1,646.99^6^, whereas MinION is free of charge because it is basically a rental from the company^7^. In this study, we did not use barcodes for MinION library preparation and performed MinION analysis using one flow cell per sample to ensure reliable data acquisition. The cost of the flow cell and library preparation reagent was $599 (for 6 reactions) and $900, respectively, resulting in MinION analysis cost of $999 per sample. If barcodes are used for library preparation, the unit cost of MinION analysis can be dramatically reduced. We usually analyze about six barcoded samples using one flow cell for bacterial species with genome sizes of around 5 Mb. If six samples are included in one analysis, the unit cost is going down to $171^8^.

The carbapenem inactivation method (CIM) and double-disk diffusion test (DDDT) are simple, inexpensive, and useful, especially in developing countries where materials and facilities are limited. In this study, the results of both tests were reasonable, as validated by subsequent genetic analysis (Figure 2). The CIM test is routinely performed at NIPH, Cambodia, and NIPH had detected and stored carbapenemase-producing bacterial isolates prior to this study. The DDDTs with SMA and CVA disks clearly detected production of MBL in *E. coli* NIPH17_0020 and ESBL in *E. coli* NIPH17_0036 (Figure 2A). CIM and DDDT are important for screening for CPGNB because MICs of carbapenems against CPGNB are not always high, as is the case for bacteria with carbapenem-hydrolyzing oxacillinase genes, such as *bla*_OXA–48_ or its variants. The MICs of carbapenems against *E. coli* NIPH17_0036, which harbored *bla*_OXA–181_ on its plasmid (Figure 4), were relatively low, whereas CIM yielded a positive result (Figure 2C). OXA-48 is capable of weakly hydrolyzing carbapenems while maintaining its activity against broad-spectrum cephalosporins. The *bla*_OXA–181_ gene is a variant of *bla*_OXA–48_, and the hydrolytic activity of OXA-181 toward β-lactams is similar to that of OXA-48 (Castanheira et al., 2011).

We sequenced carbapenemase-producing *E. coli* and *A. baumannii* isolates on-site using MinION and Bento Lab, and revealed that *E. coli* NIPH17_0020 and *E. coli* NIPH17_0036 harbored *bla*_NDM–5_ and *bla*_OXA–181_ on plasmids pNIPH17_0020_1 and pNIPH17_0036_1, respectively (Figures 3, 4) and that *A. baumannii* NIPH17_0019 harbored two copies of *bla*_OXA–23_ on its chromosome (Figure 5). Although the MinION control software MinKNOW requires a constant internet connection, the company provided us with an offline-capable version of MinKNOW. For *de novo* assembly using nanopore long-read data, we used the Miniasm software, which is fast and computationally inexpensive (Li, 2016). Because Miniasm assembles without error correction, MinION reads were error-corrected using the CLC Genomics Workbench pipeline for long-read sequencers prior to *de novo* assembly. The resultant assembly sequences obtained from on-site analysis still contained a few percent of errors that are responsible for gene frameshifts. However, the on-site *de novo* assembly sequences were sufficient for subsequent molecular epidemiological analysis (e.g., detection of AMR genes and genomic locations where the genes are encoded) (Figures 3–5 and Supplementary Figures 2, 3), and the error-corrected sequences using Illumina sequencing were subsequently used to confirm the results of the on-site analysis (Figures 3–5).

*Escherichia coli* isolates NIPH17_0020 and NIPH17_0036 belonged to ST410 according to MLST analysis. ST410 is a high-risk clone associated with AMR and recently emerged in among humans and the environment in Southeast Asia (Nadimpalli et al., 2019). In this study, both *E. coli* isolates harbored carbapenemase genes, *bla*_NDM–5_ and *bla*_OXA–181_, respectively, on their plasmids, and commonly harbored putative virulence genes, including *gad* (a glutamate decarboxylase gene), *lpfA* (a long polar fimbriae gene), and *terC* (a tellurium ion resistance gene) on their chromosomes (Supplementary Table 1). Furthermore, NIPH17_0020 harbored other virulence genes, *fyuA* (a siderophore receptor gene) and *irp2* (a siderophore gene), and NIPH17_0036 harbored other virulence gene *hra* (a heat-resistant agglutinin gene) on their chromosomes (Supplementary Table 1).

*Escherichia coli* pNIPH17_0020_1 [93.9-kb IncFIA/FIB/FII/Q1 plasmid with *bla*_NDM–5_, accession no. LC483179] was structurally highly identical to *E. coli* pPF070-NDM5 in Myanmar (accession no. AP023238) (Figure 3) and harbored several mobile gene elements (MGEs), including IS*26* and IS*CR1* surrounding *bla*_NDM–5_ (Figure 3 upper). The *bla*_NDM–5_-containing regions between IS*26* and IS*CR1* in pNIPH17_0020_1 and pPF070-NDM5 were identical with those of IncFII plasmids, such as *E. coli* pM109_FII in Myanmar (accession no. AP018139), *E. coli* pGUE-NDM in France (accession no. JQ364967), and *K. pneumoniae* pCC1409-1 in South Korea (accession no. KT725789), implying that *bla*_NDM–5_ was disseminated via plasmids and MGEs, such IS*26*, among Enterobacterales around the world (Sugawara et al., 2017).

*Escherichia coli* pNIPH17_0036_1 [51.5-kb IncX3 plasmid with *bla*_OXA–181_, accession no. LC483179] was structurally nearly identical to *E. coli* pOXA-181-IHIT35346 from Italy (accession no. KX894452), *E. coli* pOXA181 from China (accession no. KP400525), and *K. variicola* pKS22 from Thailand/Vietnam (accession no. KT005457) (Figure 4). Our analysis revealed that *bla*_OXA–181_-carrying IncX3 plasmids widespread among Enterobacterales worldwide were also present in Cambodia. pKS22 was detected in coriander imported from Thailand or Vietnam, and the international fresh vegetable trade is suspected to be a route for the spread of AMR bacteria (Zurfluh et al., 2015). Because Cambodia is geographically and culturally close to Thailand and Vietnam, it is possible for AMR bacteria to be transmitted through foods. However, this study was very small, so further analysis with larger numbers of bacterial isolates in Cambodia according to One Health approaches will be necessary to characterize AMR bacteria in this country.

*Acinetobacter baumannii* NIPH17_0019 belonging to ST571 harbored two copies of *bla*_OXA–23_ in the chromosome (Figure 5A). According to a previous study of carbapenem-resistant *A. baumannii* (Hamidian and Nigro, 2019), ST2 is the most prevalent genotype and recognized as a high-risk clone associated with AMR, and *bla*_OXA–23_ is the most widespread carbapenem-resistance gene in the world. ST571 belongs to clonal complex 2, and ST571 strains harboring *bla*_OXA–23_ are widespread in medical settings in Vietnam (Tada et al., 2015). In general, carbapenem-hydrolyzing oxacillinases hydrolyze carbapenems weakly and do not contribute to strong carbapenem resistance on their own. However, elevated expression of oxacillinase genes by the upstream insertion of IS, such as IS*Aba1* in *Acinetobacter* spp., which serves as a promoter for the downstream genes, leads to strong resistance (Turton et al., 2006; Corvec et al., 2007). Two copies of *bla*_OXA–23_ in *A. baumannii* NIPH17_0019 were located downstream of IS*Aba1* (Figure 5B). Both *bla*_oxa–23_ were present in Tn*2006*, a common 4.8-kb transposon in *Acinetobacter* app., with a central segment of 2,445-bp flanked by two reverse-oriented copies of IS*Aba1* (Nigro and Hall, 2016). Tn*2006*-containing AbaR4 is frequently inserted within the *comM* gene in the chromosome of international *A. baumannii* clones (Hamidian and Hall, 2011; Seputiene et al., 2012; Hamidian et al., 2015), and the *comM* gene is the most-preferred hotspot for AbaR insertions (Bi et al., 2019). One of the insertion sites of AbaR4 (3,924,674–3,941,483 nt region) in *A. baumannii* NIPH17_0019 was not the *comM* gene but the *mutY* gene, encoding for an adenine DNA glycosylase (Figure 5B). According to a previous study (Bi et al., 2019), AbaR insertions at *acoA*, *pho*, and *uup* genes were occasionally observed; however, *mutY* has not been previously reported as an insertion site.

## Conclusion

Based on our on-site genomic epidemiological analysis of carbapenemase-producing gram-negative bacteria in Cambodia, we revealed for the first time that plasmids and MGEs carrying clinically relevant carbapenemase genes reported in other countries have also been spreading in Cambodia. Bento Lab and MinION are useful for genomic analysis and surveillance of AMR bacteria in hospitals and research centers with limited facilities.

## Data Availability Statement

The datasets presented in this study can be found in online repositories. The names of the repository/repositories and accession number(s) can be found in the article/Supplementary Material.

## Ethics Statement

The studies involving human participants were reviewed and approved by National Ethics Committee for Health Research (NECHR), Cambodia. The patients/participants provided their written informed consent to participate in this study. Written informed consent was obtained from the individuals for the publication of any potentially identifiable images included in this article.

## Author Contributions

MS contributed to conceptualization. AH, HY, and MS contributed to methodology. MS contributed to software. AH, HY, HT, KY, and MS contributed to validation. AH and MS contributed to formal analysis, data curation, and visualization. AH, HY, HT, KY, KS, and MS contributed to investigation. PB, BW, VN, VL, MV, VA, and CD contributed to resources. AH, KS, and MS contributed to writing. CD and KS contributed to supervision. CD, KS, and MS contributed to project administration. KS and MS contributed to funding. All authors contributed to the article and approved the submitted version.

## Conflict of Interest

HY was employed by the company MicroSKY Lab., Inc., Tokyo, Japan. PB and BW were employed by the company Bento Bioworks Ltd., London, United Kingdom. The remaining authors declare that the research was conducted in the absence of any commercial or financial relationships that could be construed as a potential conflict of interest.

## Footnotes

1 https://dfast.nig.ac.jp

2 http://www.genomicepidemiology.org

3 https://galaxy.pasteur.fr/

4 http://mjsull.github.io/Easyfig/

5 http://cgview.ca

6 https://us.vwr.com/store/product/27856428/bento-lab

7 https://store.nanoporetech.com/configure-minion-basic

8 https://store.nanoporetech.com/catalog/product/view/id/226/s/rapid-barcoding-kit/category/28/

## Supplementary Material

Supplementary Figure 1 Bento Lab for on-site genomic epidemiological analysis of AMR bacteria in Cambodia. **(A)** The prototype used in this study and **(B)** current commercial type are shown.

Supplementary Figure 2 Multiple sequence alignment of carbapenemase genes analyzed by MAFFT v7.475. **(A)** Comparison between the *bla*_NDM–5_-like gene in *E. coli* pNIPH17_0020_1 (from on-site ONT analysis) and the reference gene (*bla*_NDM–5_ in accession no. JN104597), **(B)** comparison between the *bla*_OXA–181_-like gene in *E. coli* pNIPH17_0036_1 (from on-site ONT analysis) and the reference sequence (*bla*_OXA–181_ in accession no. CM004561), and **(C)** comparison between the *bla*_OXA–23_-like sequence in *A. baumannii* NIPH17_0019 (from on-site ONT analysis) and the reference sequence (*bla*_OXA–23_ in accession no. AY795964) are shown.

Supplementary Figure 3 Linear comparison of AbaR4-containing genomic regions in *A. baumannii* NIPH17_0019 harboring two copies of *bla*_OXA–23_. Two sets of AbaR4 with *bla*_OXA–23_ in *A. baumannii* NIPH17_0019: **(A)** 216,633– 233,441 nt region inserted within *comM* and **(B)** 3,924,674–3,941,483 nt region inserted within *mutY* in accession no. AP024415, and **(A,B)** comparison of both the uncorrected sequences (from on-site ONT analysis) and corrected sequences (from subsequent ONT + Illumina analysis) are shown. Red, blue, black, and gray arrows indicate carbapenemase gene (*bla*_OXA–23_), mobile gene elements (MGE), pseudogenes disrupted by AbaR4 insertion (Pseudo), and other genes (Others), respectively. The colors in comparison of sequences show percent identity.

Supplementary Table 1 Summary of whole-genome sequencing and bioinformatics analysis of AMR bacteria isolated in Cambodia in this study. Bacterial isolates, nucleotides of raw reads of MinION sequencing and the corrected reads, genome size of the uncorrected sequence (from on-site ONT analysis) and corrected sequence (from subsequent ONT + Illumina analysis) are shown. Also, AMR genes (ARG) associated with resistance to β-lactams, aminoglycosides (AG), and fluoroquinolones (FQ), virulence genes, and accession nos. of sequences are shown.
