# Supplementary figures and images for: On-Site Genomic Epidemiological Analysis of Antimicrobial-Resistant Bacteria in Cambodia With Portable Laboratory Equipment
Source: Front Microbiol. 2021 May 13;12:675463. doi: 10.3389/fmicb.2021.675463 (PMC8158813; doi:10.3389/fmicb.2021.675463)

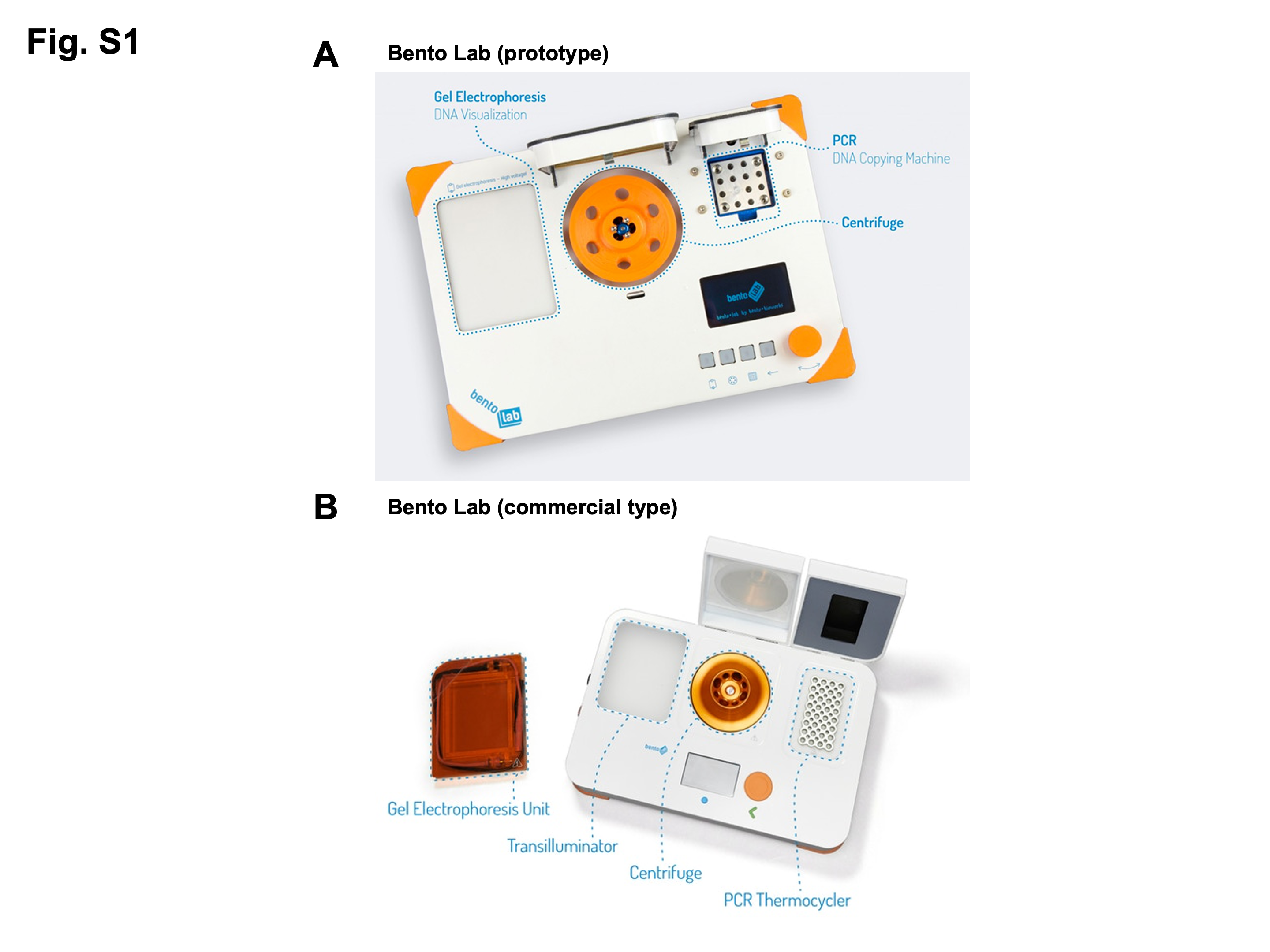

Supplement: Supplementary Figure 1 — Bento Lab for on-site genomic epidemiological analysis of AMR bacteria in Cambodia. (A) The prototype used in this study and (B) current commercial type are shown. [file Image_1.TIFF]

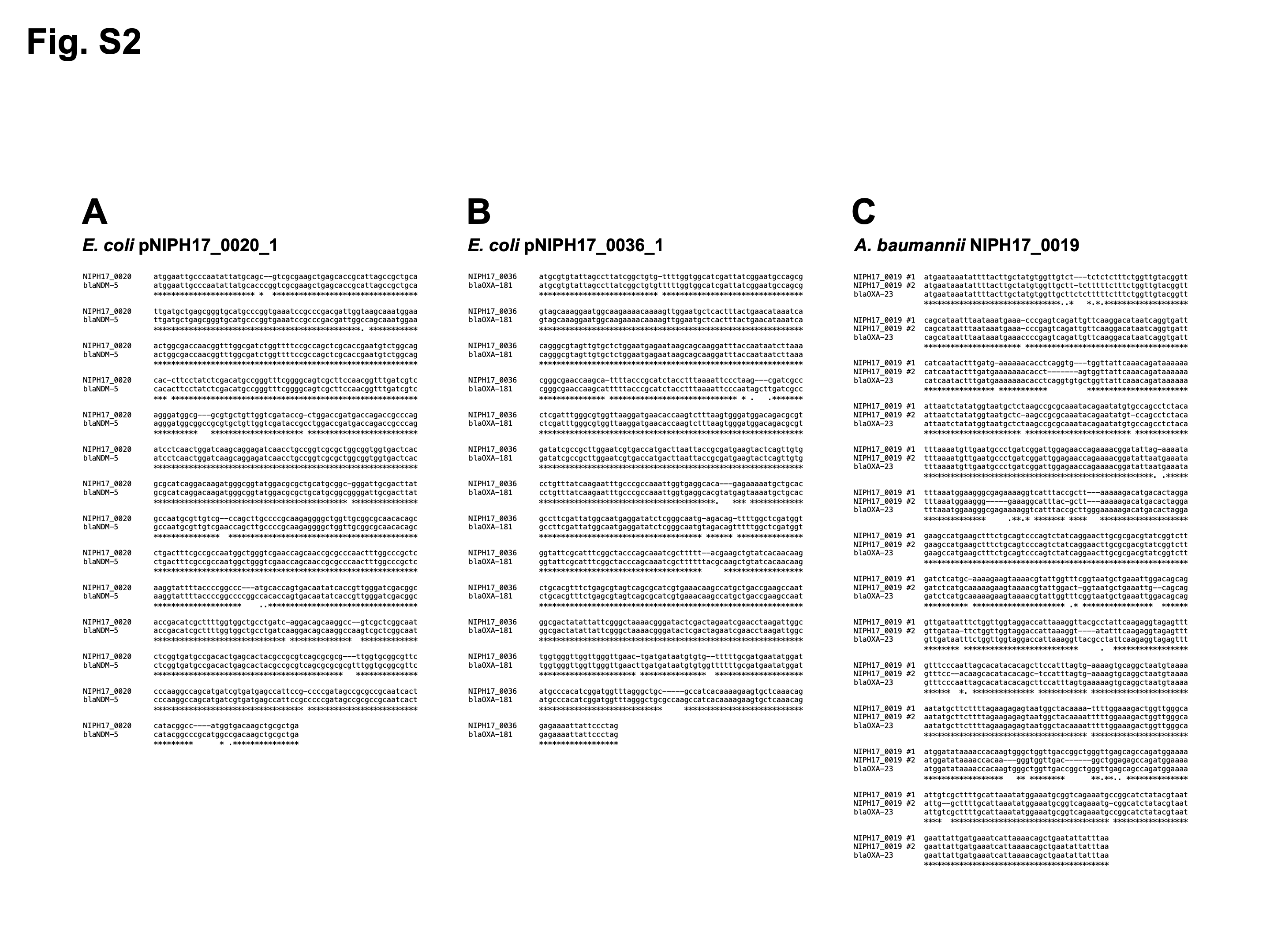

Supplement: Supplementary Figure 2 — Multiple sequence alignment of carbapenemase genes analyzed by MAFFT v7.475. (A) Comparison between the blaNDM–5-like gene in E. coli pNIPH17_0020_1 (from on-site ONT analysis) and the reference gene (blaNDM–5 in accession no. JN104597), (B) comparison between the blaOXA–181-like gene in E. coli pNIPH17_0036_1 (from on-site ONT analysis) and the reference sequence (blaOXA–181 in accession no. CM004561), and (C) comparison between the blaOXA–23-like sequence in A. baumannii NIPH17_0019 (from on-site ONT analysis) and the reference sequence (blaOXA–23 in accession no. AY795964) are shown. [file Image_2.TIFF]

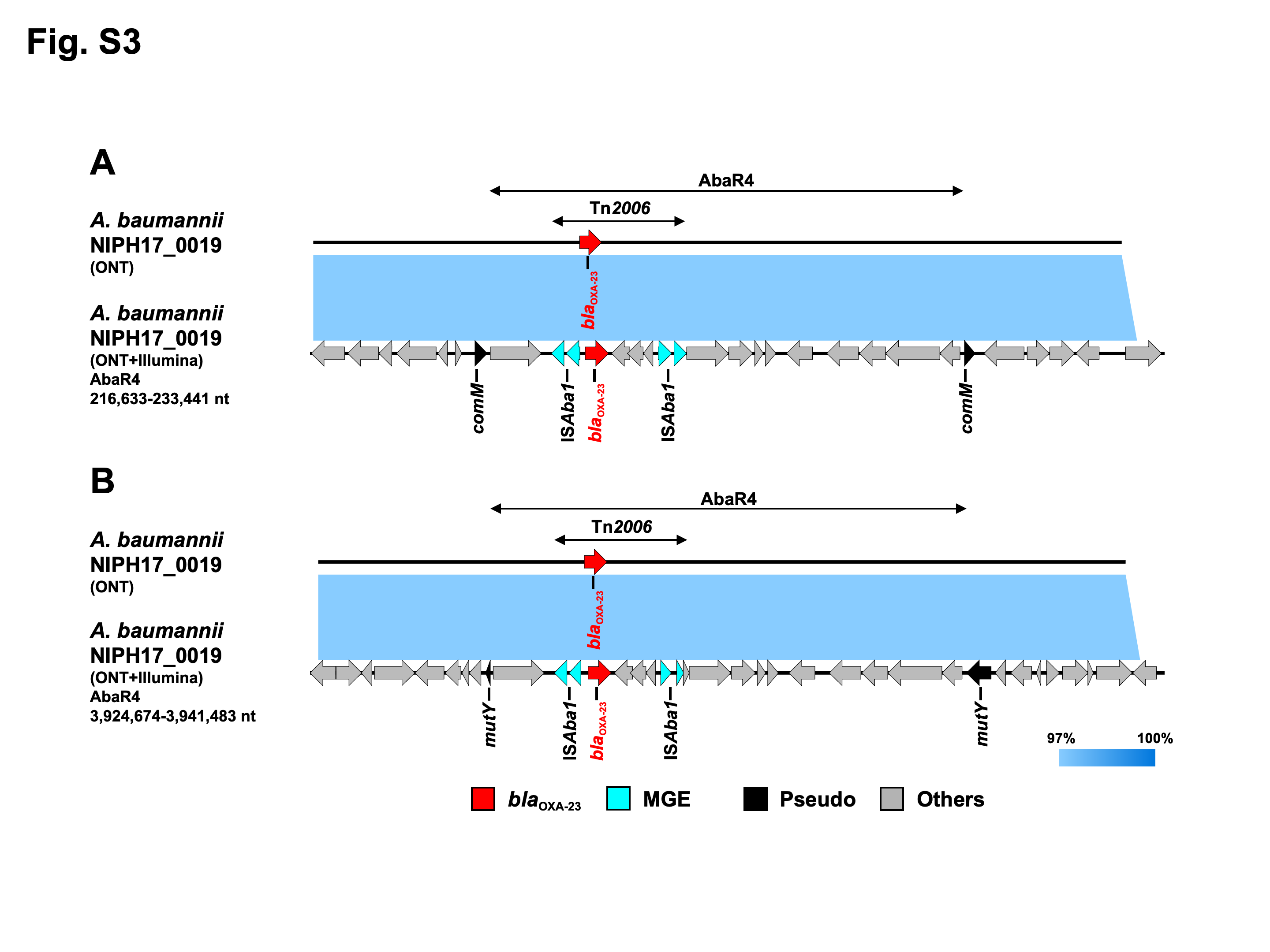

Supplement: Supplementary Figure 3 — Linear comparison of AbaR4-containing genomic regions in A. baumannii NIPH17_0019 harboring two copies of blaOXA–23. Two sets of AbaR4 with blaOXA–23 in A. baumannii NIPH17_0019: (A) 216,633– 233,441 nt region inserted within comM and (B) 3,924,674–3,941,483 nt region inserted within mutY in accession no. AP024415, and (A,B) comparison of both the uncorrected sequences (from on-site ONT analysis) and corrected sequences (from subsequent ONT + Illumina analysis) are shown. Red, blue, black, and gray arrows indicate carbapenemase gene (blaOXA–23), mobile gene elements (MGE), pseudogenes disrupted by AbaR4 insertion (Pseudo), and other genes (Others), respectively. The colors in comparison of sequences show percent identity. [file Image_3.TIFF]
