# Supplementary material for: On-Site Genomic Epidemiological Analysis of Antimicrobial-Resistant Bacteria in Cambodia With Portable Laboratory Equipment
Source: Front Microbiol. 2021 May 13;12:675463. doi: 10.3389/fmicb.2021.675463 (PMC8158813; doi:10.3389/fmicb.2021.675463)
Supplement: Supplementary Table 1 — Summary of whole-genome sequencing and bioinformatics analysis of AMR bacteria isolated in Cambodia in this study. Bacterial isolates, nucleotides of raw reads of MinION sequencing and the corrected reads, genome size of the uncorrected sequence (from on-site ONT analysis) and corrected sequence (from subsequent ONT + Illumina analysis) are shown. Also, AMR genes (ARG) associated with resistance to β-lactams, aminoglycosides (AG), and fluoroquinolones (FQ), virulence genes, and accession nos. of sequences are shown. [file Table_1.docx]

| **Isolate** | **Raw reads**  **(ONT)** | **Corrected reads (ONT)** | **Genome size**  **(ONT)** | | **Genome size**  **(ONT& Illumina)** | **ARG** | | | **Virulence**  **gene** | **Accession No.** |
| --- | --- | --- | --- | --- | --- | --- | --- | --- | --- | --- |
|  |  |  | **Contig** | **Size** | **Size** | **β-lactam** | **AG** | **FQ** |  |  |
| ***E. coli* NIPH17_0020** | **1.25 Gb** | **333.95 Mb** | **Chromosome** | **4.78 Mb** | **4.88 Mb** | ***bla*_CMY-2_** | **N.D.** | **N.D.** | ***fyuA*, *gad*, *irp2*, *lpfA*, *terC*** | **­AP024560** |
|  |  |  | **pNIPH17_0020_1**  **(plasmid)** | **91.6 kb** | **93.9 kb** | ***bla*_NDM-5_, *bla*_TEM-1B_** | ***aadA2*,  *aph(3'')-lb*, *aac(3)-lld*-like,  *aph(6)-ld*** | **N.D.** | **N.D.** | **LC483178** |
| ***E. coli* NIPH17_0036** | **1.27 Gb** | **337.27 Mb** | **Chromosome** | **4.68 Mb** | **4.78 Mb** | ***bla*_CMY-2_-like, *bla*_OXA-1_-like** | ***aadA1*-like** | **N.D.** | ***gad*, *hra*, *lpfA*, *terC*** | **­AP024561** |
|  |  |  | **pNIPH17_0036_1**  **(plasmid)** | **50.2 kb** | **51.5 kb** | ***bla*_OXA-181_** | **N.D.** | ***qnrS1*** | **N.D.** | **LC483179** |
|  |  |  | **pNIPH17_0036_2**  **(plasmid)** | **92.6 kb** | **94.8 kb** | ***bla*_TEM-217_-like, *bla*_CTX-M-15_, *bla*_OXA-1_, *bla*_TEM-1B_-like, *bla*_TEM-210_-like** | ***aac(6')-Ib-cr*,  *aac(3)-lld*-like,  *aadA5*,  *aph(6)-Id*,  *aph(3’')-Ib*** | **N.D.** | **N.D.** | **LC603215** |
| ***A. baumannii***  **NIPH17_0019** | **2.02 Gb** | **493.44 Mb** | **Chromosome** | **3.85 Mb** | **4.01 Mb** | ***bla*_OXA-23_, *bla*_OXA-23_, *bla*_OXA-66_, *bla*_TEM-1D_, *bla*_ADC-25_-like** | ***armA*** | **N.D.** | **N.D.** | **AP024415** |

**Table S1**
